# The Prevention of Heart Disease in Children

**Published:** 1903-10-03

**Authors:** J. Porter Parkinson

**Affiliations:** Physician to the North-Eastern Hospital for Children, etc.


### Hospital Clinics and Medical Progress.

### THE PREVENTION OF HEART DISEASE IN CHILDREN.

By J. Porter Parkinson, M.D., M.R.C.P., Physician to the North-Eastern Hospital for Children, etc.

A case of failing heart in a child is always a pitiful sight from its hopelessness and also from its long drawn out course of occasional temporary improvements followed by relapses. Though compensation in children is more easily established than in adults, yet the needs of the growing child demand an amount of work usually in excess of that which the damaged heart can do, and so compensation fails and the long tedious struggle begins.

In looking over statistics of hospital in-patients it would appear that in children's hospitals only about 5 per cent. of the patients are admitted for heart disease with loss of compensation, but this does not give at all a correct idea of the frequency of heart disease in children, for only the most severe cases are admitted, and many children's hospitals do not admit those beyond the age of 13, and it is after this age especially when the symptoms of loss of compensation are likely to occur.

In a case of established heart disease, cure is of course impossible, though much can be done to prolong life and render it more endurable. It is the prevention of heart disease therefore that should especially engage our attention, for there is no doubt much to be done in this direction. In considering the prevention of heart disease in children, the usual causes of such disease must be considered.

It is so well known that valvular disease may originate during illnesses such as acute rheumatism measles, scarlet fever, and chorea, that but little need be said on this head. A careful examination of the heart is frequently made in these diseases, and if any sign of developing heart complications appear, rest in the recumbent posture and other modes of treatment are always insisted upon. It is rather to the more obscure and insidious causes that I should like to allude. Rheumatism in the child occurs in such varied forms that it may be easily overlooked, especially as the joints are not often complained of. In some children attacks of tonsillitis are undoubtedly rheumatic, in others different skin eruptions, such as erythemata of various kinds, including erythema nodosum, or recurring urticaria, may be the only manifestation. Purpura is at times due to rheumatism, or rheumatic nodules may be found over subcutaneous bones or tendons. All such appearances should lead to careful examination for other evidences of rheumatism, the condition of the heart should be frequently noted, and rest insisted upon with the object of saving the heart from any unnecessary strain, and thereby lessening its tendency to inflammation. The importance of searching carefully for rheumatic nodules is exemplified in a case I saw a year or two ago; the patient was a girl aged 16, and her symptoms were exceedingly indefinite, there was no fever and no evidences of rheumatism except for one small rheumatic nodule over the extensor tendon of the thumb. Careful examination failed to reveal any others, and the heart at this time was normal; still I ventured to suggest that the patient was probably rheumatic, and later I heard from her medical attendant that a fortnight after I had seen her she was seized with joint pains and a severe attack of rheumatic fever followed, leaving her with permanent valvular disease.

It is well known how susceptible the heart of a child is to valvular disease, that a rheumatism, so slight as hardly to cause any discomfort, is able to produce endocarditis; it is, therefore, very unfortunate that we have no reliable indications of the early stages of endocarditis. The best we can do is to examine the præcordium daily, and note any alteration from the normal. We may find the apex beat more diffused, or perhaps, becoming displaced outwards, the action of the heart may be unequal or somewhat irregular. The first sound may gradually become blurred, and finally a definite murmur may appear, while the second sound may become accentuated at the apex or at the pulmonary base, or a reduplication of the second sound may be heard at the apex, and in some cases the second element of this sound gradually becomes prolonged into a diastolic murmur. The pulse-rate and character must also be noted, though in children its variations may be due to so many causes that it has not the same importance as in adults. We must remember the differences between the heart of a child and of an adult. The apex beat is usually in the fourth space and outside the nipple line till at least the fifth year of life, when it may be found in the nipple line. Again, the pulmonary second sound is normally rather louder than the aortic, and the cardiac rhythm of a child is not unfrequently irregular even in health. A systolic murmur over the right ventricle is not uncommon in healthy children, and the cardio-pulmonary murmur may also be heard.

One or more of these abnormal signs developing in a child who is or has recently suffered from some rheumatic manifestation should be the signal to confine the child rigidly to bed, in order to give the heart as much rest as possible and to allow the inflamed valve a possibility of recovery. We must also remember that endocarditis does not occur alone in children, it is probably always accompanied by myocarditis, which is an additional reason for giving the heart physiological rest.

The child should be kept in bed for several weeks after all rheumatic symptoms have disappeared. Dr. Ashby says: "It is a comparatively small matter if we are over-cautious in our treatment in keeping at rest in bed a child who has but slight joint trouble and who appears to the friends to ail little, while it is a grave matter to allow a child who is suffering from incipient endocarditis to get up and run about, or to suffer one to contract endocarditis in consequence of getting up."[1] Drugs seem of little use. A simple saline such as citrate of potash may be useful, or, if there be much fever and some pain, salicylate of soda should be given; five to ten grains may be given to children of eight years old every four hours for two or three days, and afterwards three times a day. This may be followed by a mild preparation of iron. The fact that heart disease has appeared to complicate rheumatism more frequently since the introduction of the salicylate treatment should not be put down to the drug, it is more probably due to its effect in removing pain and tenderness, which increases the difficulty of keeping patients in bed for a sufficient time to allow of the damaged heart healing and regaining its muscular strength. An ice-bag over the præcordium is recommended by some, but in a child this might lower the power of resistance and do actual harm, and it has never been shown to do real good. Flying blisters over the præcordium, or applied by Dr. Caton's method over the second, third, and fourth intercostal spaces, can do no harm, and may perhaps help to modify the inflammation.

Milk diet should be given in the cases with fever, and in milder ones arrowroot, rice, custards, eggs, etc., may be given; but soups, beef-tea, or meat should not be allowed too soon in the convalescence as they may tend to produce a relapse. After the period of strict rest in bed has passed, exercise should be resumed very gradually, and its effect on the heart noted, so that if there be any sign of over-strain more rest may be advised. Neglect of these precautions are frequently seen in both hospital and private practice. A child under my care in the North-Eastern Hospital for Children during the early part of this year was discharged, having perfectly compensated slight valvular disease with no obvious enlargement of the heart; four months later she was readmitted with cyanosis and other signs of cardiac failure, and with considerable dilatation of the heart; in the interval she had been playing and running like the other children, probably at the same time being insufficiently nourished. Rest in the hospital again caused the heart to lessen in size and the symptoms to disappear, but her condition is now serious and it is not likely she will reach the age of puberty.

This brings us to consider what mode of life ought to be suggested for those children who appear to have slight commencing heart trouble, and in whom frequent slight rheumatic incidents tend to increase the disease. In the first place it is very difficult to be quite sure of the existence of disease in some of these cases. Of course, when the heart is definitely dilated or a systolic apex murmur conducted round the side is heard, one is sure; but there are many cases where there is little but an occasional faint murmur, or alteration in character of the sounds, or some irregularity in force or rhythm of the heart beats, and these hearts are likely to puzzle and induce a different opinion in different observers. Time and experience can alone decide in such cases, and while one should hesitate to condemn a child to a semi-invalid life who has no definite sign of disease, one should also be careful not to overlook slight but genuine cases of heart affection.

Now let us suppose we are dealing with a case of slight heart disease completely compensated; our object is, of course, to maintain that compensation, and though compensation occurs more readily in the child than in the adult, yet the strain on the heart during the period of growth is proportionately more considerable than that of adult life. There are two special causes of loss of compensation, *i.e.* over-exertion and malnutrition. The first is very likely to occur in a boy at a public school, who is almost obliged to take part in football, cross-country races, rowing, etc. A moderate amount of any of these exercises may do no harm or even be beneficial, but it is the element of competition in them that leads to over-strain. The medical man attached to the school should be warned as to the necessity for supervision; at the same time one would not suggest that the child should live the life of an invalid or be deprived of the pleasure of participating in ordinary outdoor games. Also nutrition should be maintained adequately to the needs of the growing child. This, as carried out at home, is probably satisfactory, but the same cannot always be said of the public school dietary, and the defect is likely to continue while the system exists of the masters running their houses as boarding establishments for their own profit. Carbohydrates being cheap, are usually in excess, while proteids and fats such as butter are often deficient or inferior in quality. The importance of keeping up the nutrition of the heart may be judged from the fact that between the ages of 7 and 14 years the heart should double its weight, and also that proportionately to the weight of the body the heart is larger at the age of 14 than at any other age. It is important that the house be on a sandy soil, elevated above its surroundings, as there is no doubt of the effect of damp clay in predisposing to attacks of rheumatism.

Before I finally conclude I should like to mention the unfortunate difficulty which is put in the way of medical men and mothers in the case of school children of the lower classes attending the out-patient rooms of the hospital with slight rheumatism or chorea. Such cases may not be sufficiently severe to be admitted to hospital, and are undoubtedly unfit to attend school, but are sometimes constrained to do so by threats of fines, etc., by the over-zealous Board School inspectors, to the probable damage [of the heart which might otherwise have escaped injury.

[1] Ashby and Wright, "The Diseases of Children."

